# Re-Union of the Alumni of the Ohio College of Dental Surgery

**Published:** 1879-04

**Authors:** 


### Editorial.

### RE-UNION OF THE ALUMNI OF THE OHIO COLLEGE OF DENTAL SURGERY.

According to the call and arrangement previously announced in the REGISTER, the first re-union of the alumni of the Ohio College of Dental Surgery was held in the college on Thursday, March 6, at two o'clock p. m. There were about sixty of the alumni present.

A permanent organization was affected by the election of Dr. A. Berry, of the class of 1846, president.

Dr. E. G. Betty, of the class of 1876, secretary.

Dr. N. S. Hoff, of the class of 1876, treasurer.

Drs. C. H. Rosenthall, F. W. Sage and N. S. Hoff, executive committee.

After some other preliminary matters received attention, the Association adjourned to meet at half-past seven o'clock in the parlors of the Burnet House.

At the hour designated, the Alumni, and many friends and invited guests met, as per adjournment, about one hundred being present.

The assembly being called to order, the Historical Address by Dr. Geo. Watt, of the class of 1854, was read by a member of the class of 1850, Dr. Watt not being present. This was a complete history of the college from the beginning. It was replete with interesting reminescences.

By a unanimous and hearty vote the following was passed:

"The alumni of the Ohio College of Dental Surgery, present, having listened to the Historical Address by Prof. Watt, of the class of 1854, desiring to give expression to their appreciation of this very full and interesting history of their *alma mater*, and also to suitably express their respect and affection for the author, do, in this the first alumni meeting

*Resolve*, That we tender Prof. Watt our hearty thanks for the able Historical Address which he prepared for this occasion.

That we are gratified—as this paper gives abundant evidence—that although enfeebled in body, he still retains that mental vigor and facility as a writer, which has justly distinguished him as a member of the dental profession. That we desire also to give expression to our recognition and appreciation of the substantial work Prof. Watt has accomplished in the advancement of dental science, education and literature during the past twenty years, and to heartily wish that he may yet be spared many years to his family and friends."

After this Dr. Jas. Taylor gave some interesting personal reminiscences of dentistry in this country; he also gave some interesting financial statistics of the college.

Reports of the classes, in the order of graduation from 1846, to 1860, so far as they were represented, was made. These were interesting and called up thoughts of by gone days and pleasant memories of those who were absent. Tender mention was made of quite a number who have passed from the scenes and labors of this life, and the expressions given of such indicate that their names will not soon pass from the memories of their class mates.

The reports of the classes subsequent to 1860 are to be given at the next annual meeting.

At this stage of the proceedings the large dining room of the house was thrown open, and the members and guests, without much special urging, entered and took seats at two tables, which were fully ladened with all the good things which the hosts of the Burnet know so well how to provide. If this occasion is an index, no one ever goes from this House empty or dissatisfied.

After the tables had been relieved of their burden, so far as it could be done by those present, the following toasts were presented and responses made:

"*The Dental Profession.*" Response by Dr. W. H. Morgan.

"*Dental Education.*" Response by Dr. A. O. Rawls.

"*Our Alumni in other Lands.*" Response by Dr. J. Taft.

"*The Hope of the Profession.*" Response by Dr. Geo. W. Keely.

"*The Brotherhood of the Professions.*" Response by Dr. J. H. Prewitt.

"*The Ohio Dental College.*" Response by Dr. H. A. Smith.

Volunteer toasts being in order, Drs. Taylor, Clendenin and Cilley responded to calls. Some of these responses were so full of side splitting humor, that it is unsafe to publish a report of them, and so we omit.

After organizing in the parlors, the Association adjourned to meet on the first Thursday in March, 1880, at half-past seven o'clock p. m.

May this meeting only be a beginning of what shall be.

